# ^1^H-NMR-Based Metabolomic Profiling of CSF in Early Amyotrophic Lateral Sclerosis

**DOI:** 10.1371/journal.pone.0013223

**Published:** 2010-10-08

**Authors:** Hélène Blasco, Philippe Corcia, Caroline Moreau, Ségolène Veau, Clémentine Fournier, Patrick Vourc'h, Patrick Emond, Paul Gordon, Pierre-François Pradat, Julien Praline, David Devos, Lydie Nadal-Desbarats, Christian R. Andres

**Affiliations:** 1 Inserm U930, CNRS 2448, Tours, France; 2 Université François-Rabelais, Tours, France; 3 CHRU de Tours, Laboratoire de Biochimie et Biologie Moléculaire, Tours, France; 4 CHRU de Tours, Service de Neurologie, Tours, France; 5 CHRU de Lille, Service de Neurologie, Lille, France; 6 Laboratoire de RMN - PPF “Analyses des Systèmes Biologiques”, Université François-Rabelais, Tours, France; 7 Northern Navajo Medical Center, Shiprock, New Mexico, United States of America; 8 APHP, Fédération des Maladies du Système Nerveux, Centre Référent Maladie Rare SLA, Hôpital de la Pitié-Salpétriere, Paris, France; Indiana University, United States of America

## Abstract

**Background:**

Pathophysiological mechanisms involved in amyotrophic lateral sclerosis (ALS) are complex and none has identified reliable markers useful in routine patient evaluation. The aim of this study was to analyze the CSF of patients with ALS by ^1^H NMR (Nuclear Magnetic Resonance) spectroscopy in order to identify biomarkers in the early stages of the disease, and to evaluate the biochemical factors involved in ALS.

**Methodology:**

CSF samples were collected from patients with ALS at the time of diagnosis and from patients without neurodegenerative diseases. One and two-dimensional ^1^H NMR analyses were performed and metabolites were quantified by the ERETIC method. We compared the concentrations of CSF metabolites between both groups. Finally, we performed principal component (PCA) and discriminant analyses.

**Principal Findings:**

Fifty CSF samples from ALS patients and 44 from controls were analyzed. We quantified 17 metabolites including amino-acids, organic acids, and ketone bodies. Quantitative analysis revealed significantly lower acetate concentrations (p = 0.0002) in ALS patients compared to controls. Concentration of acetone trended higher (p = 0.015), and those of pyruvate (p = 0.002) and ascorbate (p = 0.003) were higher in the ALS group. PCA demonstrated that the pattern of analyzed metabolites discriminated between groups. Discriminant analysis using an algorithm of 17 metabolites revealed that patients were accurately classified 81.6% of the time.

**Conclusion/Significance:**

CSF screening by NMR spectroscopy could be a useful, simple and low cost tool to improve the early diagnosis of ALS. The results indicate a perturbation of glucose metabolism, and the need to further explore cerebral energetic metabolism.

## Introduction

Amyotrophic lateral sclerosis (ALS), the most common adult-onset motor neuron disease, is characterized by degeneration of both lower and upper motor neurons leading to death within 2–5 years of onset [1]. Although the underlying causes of motor neuron degeneration are still unknown, hypotheses suggest a role for oxidative stress, mitochondrial dysfunction, glutamate-mediated excitotoxicity, cytoskeletal abnormalities, and protein aggregation [2].

The lack of biological tools to diagnose ALS together with the non specificity and heterogeneity of clinical symptoms lead difficulty in making the diagnosis in very early stages. Indeed, the time between the appearance of first symptoms and the diagnosis is often 9 months or more [3]. Several authors have studied the potential use of blood and cerebrospinal fluid (CSF) biomarkers associated with ALS but these studies were limited to small numbers of target molecules supposed to be linked to ALS pathogenesis [4], [5], [6]. While many *in vivo* studies have provided clues to pathogenesis mechanisms, none has identified reliable markers useful in routine patient evaluation. Recently, high-throughput techniques such as metabolomics have been used to evaluate a combination of markers in patients with neurological diseases [7]. Metabolomic studies have been performed via different analytical methods such as high performance liquid chromatography followed by electrochemical detection [8] or high resolution ^1^H NMR (Nuclear Magnetic Resonance) spectroscopy [9]. NMR spectroscopy appears to be cost-effective, useful in routine care, and screening [10]. Different kinds of biological fluids have been screened [11], but CSF may have the highest yield of biomarkers in ALS because of its direct contact with the brain, its accessibility, and its dynamic changes with the cerebral environment.

The aim of this study was to search for a metabolic signature of ALS in the CSF using high resolution NMR spectroscopy. We compared ^1^H NMR spectra of CSF samples collected from ALS patients and from patients without a neurodegenerative disease.

## Methods

### Patients and controls

Cerebrospinal fluid samples were obtained at the time of diagnosis from patients in three French ALS centers (Tours, Lille and Paris). The diagnosis of ALS was made according to diagnostic criteria for definite or probable ALS based on the El Escorial World Federation diagnostic criteria [12]. In routine practice, a CSF analysis is performed in all patients in whom a diagnosis of ALS is suspected. All patients involved in our study gave informed consent. Patients from Lille and Paris gave written consent to perform research with their CSF samples. In routine practice, a CSF analysis is performed in all patients in whom a diagnosis of ALS is suspected. For our study, we used a part of CSF sample to perform complementary analysis with NMR. In Tours, before starting this study, we asked our local ethics committee about the requirement of informed consent. We obtained verbal permission from the Persons Protection Committee's President of Tours to use CSF samples obtained by routine check up and without written consent of patients and controls. Patients from Tours gave verbal consent to use their CSF samples for medical research. One of the clinicians was responsible to give to patients the appropriate information about the use of their CSF samples in medical research. Ethics committees approved consent procedure.

For each patient, information on diagnosis, gender, age, site-of-onset and age-at-onset were obtained. The site-of-onset was defined as either bulbar or limb-onset. The age-at-onset was defined as the time at which motor weakness was first noted by the patient. We compared clinical, demographic data and biological parameters of ALS patients between each center. According to ethical considerations, it is not conceivable to collect CSF from healthy subjects. Then, the control group encompassed individuals with non-neurodegenerative diseases having a routine lumbar puncture at the time of diagnosis. Standard clinical laboratory tests including bacteriological, biochemical analyses of CSF and blood glucose levels were collected for each subject.

### Sample preparation

CSF samples were stored in polypropylene tubes at −80°C immediately after collection and until analysis [13]. Before the NMR experiments, samples were thawed, and centrifugated at 3000g for 5 minutes. Then 0.5 mL of CSF were mixed with 0.1 mL of deuterium oxyde solution to calibrate the NMR spectrometer. Final adjustment of pH to 9.5±0.15 was done with HCl or NaOH solutions using a pH-meter equipped with a glass combination electrode. Finally, 0.6 mL of the prepared solutions were transferred to a 5-mm NMR tube (CortecNet, Paris, France) for ^1^H NMR analysis.

### Magnetic Resonance Spectroscopy experiments

The ^1^H NMR spectra were performed on a Bruker DRX-500 spectrometer (Bruker SADIS, Wissembourg, France), operating at 11.7 T, with a Broad Band Inverse (BBI) probehead equipped with Z gradient coil. NMR measurements were done at 298K, with non-spinning samples. CPMG ^1^H NMR spectra were recorded with a spin-spin relaxation delay of 80ms (echo time 200 µs repeated 200 times), collected with 128 transients, eight dummy scans and a d1 of 10s into 32k data points with a spectral width of 7500 Hz and an acquisition time of 2.04s. The water peak was irradiated during d1 delay. Unambiguous assignments were performed using a two-dimensional correlation spectroscopy. Eight transients per increment and 256 increments were collected into 4K data points. COSY spectra were acquired with 3s relaxation delay, 6000Hz spectral width in both directions. The water signal was irradiated during the recycling time. Shimming of the sample was performed automatically on the water signal. For quantification of metabolite peaks, the electronic reference (ERETIC) signal was set during acquisition time [14]. The ERETIC pulse was generated by a second radio frequency channel phase-synchronized with the NMR signal of the sample. ERETIC intensity, frequency and line broadening were calibrated with a reference tube to determine metabolite concentrations.

Data were processed using XWinNMR version 3.5 software (Bruker Daltonik, Karlsruhe, Germany). Prior to Fourier transformation (FT), the 1D FIDs were zero-filled to 64K data points which provided sufficient data points for each resonance, and a line broadening factor of 0.3Hz was applied. All spectra were corrected for phase distortion and the baseline was manually corrected for each spectrum. Each spectrum was integrated using WinNMR software integral function.

Quantification of metabolite peaks was performed with the ERETIC peak as a quantitative reference. Calibration of the ERETIC peak, which had the same area in all spectra, was made using a mixture of 9.13mM of citrate, 9.20mM of lactate, and 9.10mM of glutamate as the reference tube. According to this reference tube, the measured area of the ERETIC peak (A_E_) symbolized a calculated concentration (C_E_) of 4,67mM. Metabolite concentration (C_X_) was calculated according to the following formula:
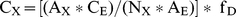
where Cx is the metabolite concentration in the CSF sample, Nx is the number of protons of the quantified peak metabolite, C_E_ is the calculated concentration for the ERETIC peak, A_E_ is the ERETIC area peak, and f_D_ is the dilution factor of the CSF sample.

Spectral ^1^H assignments were achieved according to the literature values of chemical shifts in various media and biofluids [15]. Because beta-methyl-L-amino-alanine (BMAA) has been recently reported by several authors as potentially involved in the etiology of ALS, we searched for its presence [16].

### Statistical analysis

#### Univariate data analysis

A comparison of CSF concentrations between ALS and non ALS patients was done to highlight the potential disturbances in the metabolic pathway due to ALS.

We compared concentrations of CSF metabolites between ALS and non ALS patients using t-tests or Wilcoxon tests. Among the 53 metabolites identifiable in CSF by NMR [17], we quantified (by XWin NMR software) those having the highest signal, i.e quantifiable with the highest accuracy.

A correction for multiple tests was applied to adjust the p values by accounting for the 17 metabolites evaluated in the analysis. Differences were considered as significant when p<0.003.

Statistical analysis was performed with JMP statistical software version 7.0.2 (SAS Institute, Cary, North Carolina).

#### Multivariate data analysis

Principal component analysis (PCA) was performed using SPAD^©^ software (SPAD v5.6 software decisia, France). PCA is a descriptive multivariate analysis that rapidly identifies groups of samples according to their scores in different variables (i.e. different metabolites). Spectral variation is reduced to a series of principal components (PC), each representing correlated spectral changes and summarized in a score plot. PCs are new variables that are orthogonal to each other and explain progressively less variance in the data set. PCs were displayed in a two- dimensional score plot, allowing visualization of the distribution and grouping of the samples in the new variable space.

Discriminating analysis was performed to evaluate how closely a set of measurement metabolites predict the classification of ALS and non ALS patients. Thus, we randomly divided our data into a training set of size 50 and a test set that consisted of the remaining patients. The model was built based on the training set with known clinical status to construct a set of linear functions of the measured metabolites. The clinical status was predicted from the test group to evaluate the power of prediction.

Statistical analysis was performed with JMP statistical software version 7.0.2 (SAS Institute, Cary, North Carolina) and R version 2.8.0, an open source program developed by the R Foundation for Statistical Computing (Vienna, Austria).

## Results

### Patients and controls

Clinical data and CSF samples were obtained from 50 patients with ALS (22 from Tours, 19 from Lille and 9 from Paris) and 44 controls. No differences in clinical, demographic or biological data were found between patients from Tours, Lille and Paris, so we pooled these samples into a single group: the ALS group. 61% of ALS patients were male and had median age-at-onset of 62.5 years (22.9–90.6). 65% of ALS patients had limb-onset. The control group encompassed patients with the following diseases : sarcoidosis (one patient), psychiatric disorders (three patients), cerebellar syndrome (one patient), peripheral neuropathy (thirteen patients), chronic inflammatory demyelinating polyneuropathy (six patients), muscular cramps (one patient), facial paralysis (one patient), traumatic cerebral injury (one patient), ataxia with axonal neuropathy (one patient), normal pressure hydrocephalus (two patients), paresthesia (two patients), epileptic seizures (one patient), spinal cord infarction (one patient), cervical spondylotic myelopathy (three patients), adrenomyeloneuropathy (one patient), non-organic hypoesthesia (one patient), neuroborreliosis (one patient), dystonia (one patient), Hallervorden-Spatz syndrome (one patient), non neurologic deafness (one patient), and oculomotor paralysis (one patient). Controls were matched with ALS patients by age and sex. There were no differences in standard bacteriological and biochemical tests of CSF, or in blood glucose levels between groups (**Table 1**).

**Table 1:** ALS and non ALS patients characteristics.

	ALS patients (n = 50)	non ALS patients (n = 44)	p
**Sex**			
Male	31 (62%)	27 (61%)	1.00
Female	19 (38%)	17 (39%)	
**Age**	64.6 (35.0–90.6)	60.6 (26.8–82.9)	0.20
**Blood**			
Glucose (mmol/L)	5.3 (4.4–7.0)	5.9 (4.8–9.0)	0.11
**LCR**			
Leucocytes/mm^3^	1 (0–50)	1 (0–75)	0.32
Cell/mm^3^	0 (0–2200)	1 (0–75)	0.80
Protein (g/L)	0.43 (0.33–1.08)	0.45 (0.24–6.23)	0.89
Glucose (mmol/L)	3.3(0.56–7.16)	3.2 (0.15–8.9)	0.51

Data are expressed in median (range).

### Qualitative ^1^H-NMR analysis and identification

**Figure 1:**
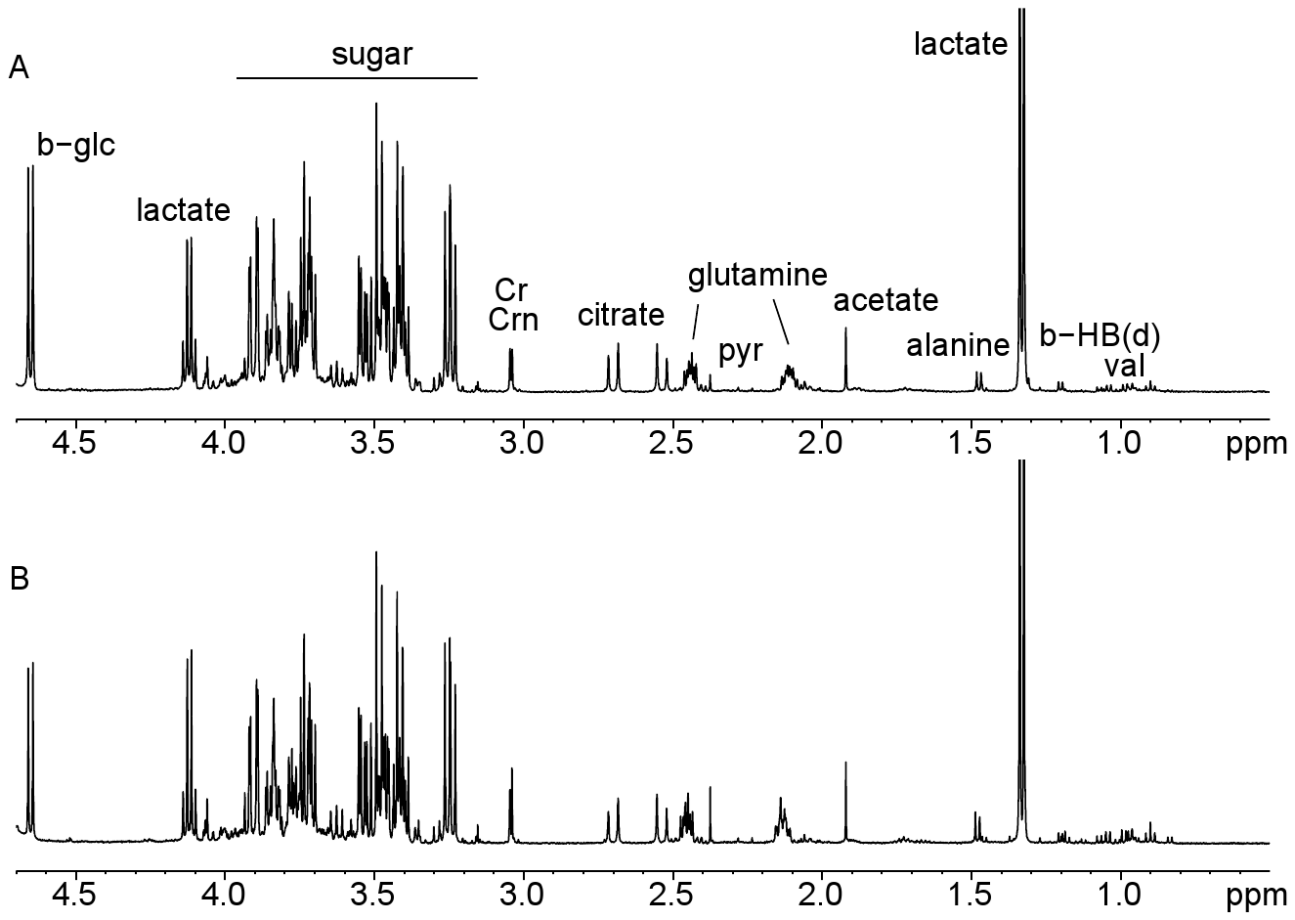
CSF spectra by ^1^H NMR from ALS patient (A) and non ALS patient (B).

The ^1^H-NMR signals of all common metabolites including amino-acids, organic acids, and carbohydrates were assigned according to previous publications. [17]. Qualitative analysis of CSF by superimposition of spectra from ALS patients and controls did not reveal any differences in metabolite composition.We found particularly no trace of BMAA in the CSF of our ALS patients. Accurate visual comparison of the spectra was difficult because of dilution factor. An example of CSF spectra from a typical ALS patient and control are shown on **figure 1**.

### Univariate data analysis

We analysed 17 CSF metabolites in ALS and non ALS patients, defined as follows: amino-acids (alanine, glutamine, tyrosine), organic acids (citrate, acetate, α-hydroxybutyrate (AHBT)), ketone bodies (β-hydroxybutyrate (BHBT), acetone, acetoacetate), glucose, fructose, metabolites involved in glucose metabolism (pyruvate, lactate, creatinine and creatine, recently identified as markers of mitochondrial dysfunction [18], the anti-oxidant molecule ascorbate, and formate as well as ethanol.

**Figure 2:**
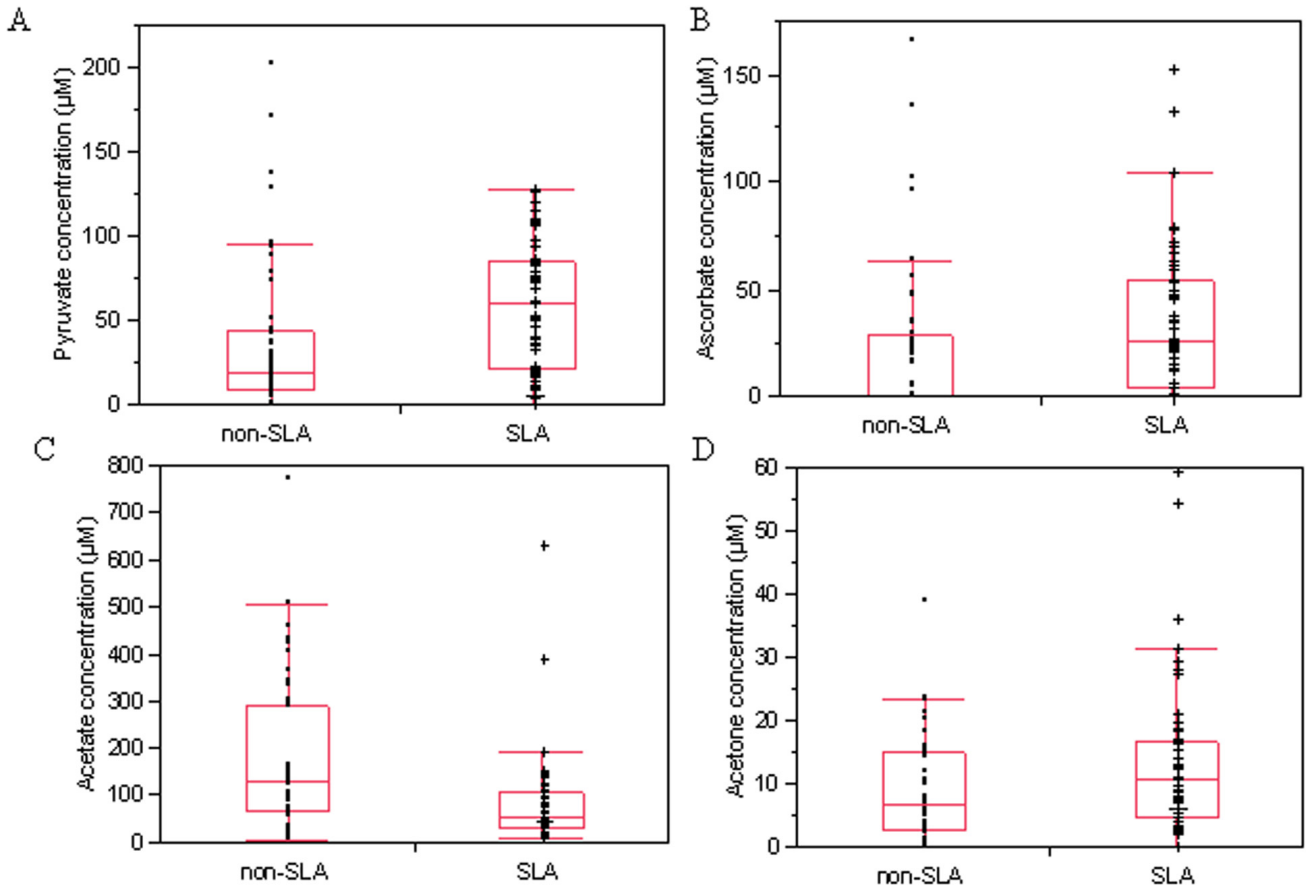
Concentrations of different metabolites in ALS (n = 50) and non ALS patients (n = 44). CSF concentrations of pyruvate (A), ascorbate (B), acetate (C) and acetone (D) are represented with median, upper and lower quartile, minimum and maximum data values.

Results of quantitative analyses of metabolites in CSF from ALS patients and controls are presented in **table 2**. We noted 3 metabolites for which mean CSF concentrations were significantly different between groups (p<0.003). We found significantly lower concentrations of acetate in ALS patients than in controls (53 vs 104 µmol/L, p = 0.0002). There were higher concentrations of ascorbate and pyruvate in ALS patients compared to non ALS patients (p = 0.003 and p = 0.002, respectively). The lactate/pyruvate ratio, a marker of cytosolic oxido-reduction state was lower in ALS patients than in controls (56 vs 115, p = 0.0029). Acetone concentrations trended to be higher in ALS patients (p = 0.015). Box plots representing acetate, acetone, ascorbate, and pyruvate concentrations in the ALS and non ALS groups are shown in **figure 2**.

**Table 2:** CSF metabolite profiles in ALS and control subjects measured by ^1^H NMR spectroscopy.

Metabolites (µmol/L)	ALS (n = 50)	Non ALS (n = 44)	p
AHBT	61 (0–137)	59 (0–177)	0.17
Ethanol	24 (0–170)	24 (0–460)	0.29
Alanine	56 (6–596)	61 (0–214)	0.74
Acetate	53 (9–389)	104 (2–366)	**0.0002**
BHBT	17 (4–85)	27 (0–134)	0.97
Lactate	2076 (444–3892)	2020 (94–5322)	0.81
Acetoacetate	9 (0–29)	8 (0–29)	0.89
Acetone	11 (0–59)	5 (0–23)	0.015
Glutamine	716 (139–1272)	785 (35–1296)	0.24
Pyruvate	68 (3–128)	17 (0–201)	**0.002**
Citrate	283 (22–49)	285 (12–754)	0.51
Creatine/creatinine	155 (25–316)	160 (9–450)	0.65
Glucose	3240 (560–7166)	3174 (150–8928)	0.98
Fructose	428 (0–1224)	419 (0–967)	0.82
Ascorbate	25 (0–152)	0 (0–165)	**0.003**
Tyrosine	12 (0–48)	8 (0–117)	0.35
Formate	28 (3–91)	30 (1–187)	0.35

Data are expressed in median and range. P value inferior to threshold after correction for multiple test (p = 0.003) are shown in bold.

AHBT: Alphahydroxybutyrate.

BHBT: Betahydroxybutyrate.

### PCA of the ^1^H-NMR metabolite differences between ALS and non-ALS patients

**Figure 3:**
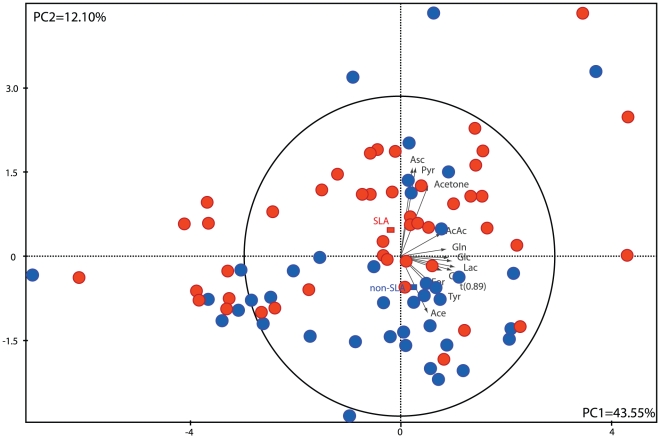
Principal Component Analysis score plot for ALS (n = 50) and non ALS patients (n = 44).

PCA analysis was applied to the NMR data using the 17 metabolites integrated in all patients. The resulting PCA score plot is shown in **figure 3**, with the two first PCs explaining 55.65% of the variation in the selected metabolites. The PCA scores plot for the quantitative NMR data showed separated clusters for ALS and non ALS patients. Each spectrum can be viewed as an observation in PCA space where the proximity of observations represents the similarity of the metabolic profiles of CSF samples. The PCA score plot shows the metabolites contributing to the separation of the two clusters and allows the identification of metabolites acting on this clustering. The ALS patients seemed to be characterized by high concentrations of pyruvate, ascorbate, and acetone whereas the non-ALS group seemed to be weighted by lower concentrations of these metabolites and by high concentrations of formate, acetate and β-hydroxybutyrate.

All metabolites were entered into the discriminant model. According to this panel of metabolites, we evaluated the clinical status of patients and identified that they were predicted in the correct group with a probability evaluated at 81.6%.

## Discussion

Metabolomic studies are a powerful approach to ascertaining metabolic signatures from a combination of small molecules in biological fluid and could lead to the identification of diagnostic or prognostic biomarkers. NMR spectroscopy is a non-invasive technique, needs little sample preparation and gives an overview of the principal metabolic pathways. To our knowledge, the present study is the first to address the potential use of ^1^H NMR spectroscopy in analysing CSF from ALS patients.

We compared CSF metabolic profiles between ALS and non-ALS patients. First, we conducted a qualitative analysis to measure any qualitative difference between groups. Among the 17 metabolites measured in our study, 13 were also analysed by Kumar et al. in serum samples of ALS patients [9], and we detected the same metabolites and in the same range as that reported in a previous study of human CSF metabolome [17]. Importantly, glutamate could not be detected in CSF of patients and controls by NMR technique. Fiszmann in a recent study [19] found a glutamate concentration around 0.6–0.9 µM in control CSF and 2.6–6.1 µM in ALS CSF. Even if there may be an increase of glutamate in ALS patients, the NMR methodology is not able to pick up such very low concentrations.

Univariate analysis showed higher concentrations of CSF ascorbate in ALS patients. Two previous studies have compared concentrations of ascorbate between ALS patients and controls but results were contradictory [20]–[21]. The elevated levels of this anti-oxidant molecule are compatible with oxidative stress previously described in ALS and could also be linked to ascorbic acid release from astrocytes after glutamate stimulation [22], [23]. Glutamate-mediated excitotoxicity is a recognized factor in the pathophysiology of ALS and could result in elevated levels of ascorbic acid. Ascorbic acid might, by itself, modulate neuronal metabolism through inhibition of glucose consumption during episodes of glutamatergic synaptic activity and by stimulating lactate uptake in neurons [23], [24], observations consistent with the lower lactate/pyruvate ratio seen in our ALS patients. These findings together with data previously published on metabolic modifications suggest a perturbation of brain glucose metabolism in patients with ALS [11].

We also noted lower CSF concentrations of acetate and a trend toward higher CSF concentrations of acetone in ALS patients. We found no differences in other ketone bodies between ALS patients and controls, but did note high interindividual variability of these parameters. Kumar et al. described higher concentrations of ketone bodies (BHBT and acetone) and acetate in the serum of ALS patients [9]. Based on the energetic metabolism in brain compared to the blood compartment, Kumar's results do not necessarily contradict our own. Hypermetabolism described in ALS patients may lead to increased energy requirements in muscle and brain [25], [26]. Consequently, in addition to the usual energetic substrates (carbohydrates, lactate, fat), ketone bodies and acetate could also be used as a source of energy in the brains of ALS patients [27], [28]. Thus the high levels of ketone bodies and acetate reported in serum could be due to the increased beta oxidation of fatty acids, related to higher energy needs [9], [29]. Monocarboxylic acid transporters (MCT1) mediate the transport of ketone bodies and acetate from blood to brain through the Blood Brain Barrier (BBB) and their influx into the brain is largely determined by the amount of ketones and acetate present in the blood [27], [30]. Thus, acetate, acetoacetate and β-hydroxybutyrate may be consumed by neurones and astrocytes to provide energy. However, metabolism of acetone is different than for the other ketones, perhaps explaining the still moderately higher level of acetone in ALS patients [31].

Based on these data, we used two statistical approaches to assess diagnostic consistency in ALS.

First, we performed the multivariate analysis PCA on the 17 metabolites detected by ^1^H NMR spectroscopy to provide a metabolic signature for ALS. The scatter plot of PCA results derived from the NMR spectra discriminated between ALS and non-ALS populations. Of the metabolites responsible for the observed separation on the PCA score plot, we found the same discriminating metabolites as found using univariate analysis.

A discriminant analysis was performed to predict the classification of ALS and non-ALS patients. The predictive power was superior to 80%, sufficient to consider this panel of metabolites useful in clinical practice. To our knowledge, Rozen et al. [8] were the first and the only investigators to conduct a metabolomic study using a multivariate approach. They studied perturbations of the blood metabolome of 28 patients with motor neuron diseases (MND) and 30 healthy controls using high performance liquid chromatography. Of over 317 metabolites, they identified 50 that were elevated in MND patients and 70 that were decreased (p<0.05). Similar to our study, they separated MND patients from controls using multivariate regression techniques.

This study demonstrates that ^1^H NMR spectroscopy combined with multivariate analysis can detect changes in the concentrations of some CSF metabolites in ALS, and highlights specific metabolic pathway perturbations. Moreover to our knowledge, this study is s the first to provide a diagnostic predictive power better than 80%. However, the composition of CSF may not be the direct consequence of metabolism in neurones and astrocytes, due in part to the role of other independent factors such as BBB transporters and release of metabolites from endothelial cells. Despite the invasiveness of lumbar puncture, the benefits of a correct diagnosis based on CSF component analysis may outweigh the risk of the puncture procedure. [32] Future studies may reach improved sensitivity and specificity by comparing blood and CSF metabolites and discriminating the disturbances of metabolism in blood from brain.

To our knowledge, this study is the first one reporting in CSF the use of ^1^H NMR spectroscopy to explore metabolic pathways involved in the pathogenesis of ALS. Our data expand on previous studies and show promise that a biomarker panel could be developed for helping in the early diagnosis of ALS. Further studies with larger numbers of patients and controls, including other motor neuron diseases, will be crucial to validate this model and to assure its place in routine practice. It is also necessary to valid this panel of biomarkers in another cross-sectional study with a separate population, and of interest to perform a longitudinal study to determine predictive power of this model on disease progression.

A better understanding of heterogeneous genetic, biochemical and clinical features of ALS may help to define pathological profiles among ALS patients. This study focuses on the NMR profile, but we are aware that accuracy of early diagnosis of ALS will depend on combination of several approaches like imaging, electrophysiological and biological markers.
