# Correction: ^1^H-NMR-Based Metabolomic Profiling of CSF in Early Amyotrophic Lateral Sclerosis

**DOI:** 10.1371/annotation/2c2f8fce-a5be-40a3-af8f-48f119b2c593

**Published:** 2010-11-10

**Authors:** Hélène Blasco, Philippe Corcia, Caroline Moreau, Ségolène Veau, Clémentine Fournier, Patrick Vourc'h, Patrick Emond, Paul Gordon, Pierre-François Pradat, Julien Praline, David Devos, Lydie Nadal-Desbarats, Christian R. Andres

The following information was missing from the Funding section: This study was funded by INSERM and Université François Rabelais de Tours. 

